# Conservative Management of Deep Residual Periodontal Pockets Using Adjunctive Mucoadhesive Polymeric Hydrogel: A Case Report With 4‐Month Follow‐Up

**DOI:** 10.1155/crid/3170326

**Published:** 2026-07-08

**Authors:** Stefano Sarri, Scilla Sparabombe, Riccardo Scaringi, Enrico Fiorini, Lorenzo De Marco, Simone Sevi

**Affiliations:** ^1^ Independent Researcher, Monza, Brianza, Italy; ^2^ Department of Medicine and Surgery, Polytechnic University of Marche, Ancona, Italy, univpm.it; ^3^ Independent Researcher, Milan, Italy; ^4^ Department of Medicine and Surgery, University of Insubria, Varese, Italy, uninsubria.eu

**Keywords:** case report, furcation involvement, mucoadhesive hydrogel, periodontitis, residual periodontal pocket

## Abstract

**Background:**

Deep residual periodontal pockets after nonsurgical periodontal therapy represent a clinical challenge, particularly in molar sites with furcation involvement. This case report describes the short‐term outcome of a combined conservative retreatment protocol including repeated subgingival instrumentation, erythritol‐based air polishing, and adjunctive application of a mucoadhesive polymeric hydrogel.

**Case Presentation:**

A 57‐year‐old systemically healthy, nonsmoking female with periodontitis, Stage III Grade B, presented with a persistent 9 mm residual pocket and Grade II buccal furcation involvement at Tooth 26 (maxillary left first molar) after initial nonsurgical periodontal therapy. The patient showed good plaque control and preferred to avoid surgical treatment. The site was retreated with mechanical debridement, subgingival erythritol air polishing, and adjunctive hydrogel application. A second hydrogel application was performed after 37 days because bleeding on probing persisted despite partial pocket reduction.

**Outcome:**

At 4 months after the first hydrogel application, probing depth at the treated site decreased from 9 to 3 mm, bleeding on probing was absent, and furcation involvement was clinically reduced from Grade II to Grade I. No adverse events were reported.

**Conclusions:**

In this single highly compliant patient, a combined conservative retreatment protocol was associated with short‐term clinical improvement of a deep residual periodontal pocket with furcation involvement. Because multiple interventions were performed and concurrent clear‐aligner therapy was present, the specific contribution of the hydrogel cannot be isolated. Longer follow‐up and controlled studies are needed before any inference regarding efficacy or equivalence to surgical therapy can be made.

## 1. Introduction

Periodontitis is a multifactorial inflammatory disease characterized by progressive destruction of the periodontal supporting tissues and driven by dysbiotic microbial communities and host‐response dysregulation [1, 2].

Residual periodontal pockets after nonsurgical periodontal therapy remain clinically relevant because they may represent sites of persistent inflammation and increased risk for future disease progression [3, 4]. This issue is particularly important in molar regions, where complex root anatomy, furcation involvement, and limited access for instrumentation may reduce the predictability of subgingival debridement [5, 6]. Although subgingival instrumentation combined with effective plaque control is the foundation of periodontitis treatment, localized sites may show incomplete clinical response despite overall improvement and good patient compliance [4].

Adjunctive local delivery systems have been proposed to support periodontal healing after mechanical biofilm disruption [7, 8]. Among these, mucoadhesive polymeric hydrogels are designed to remain within the periodontal pocket and to provide local delivery of bioactive components after instrumentation [9–12]. A randomized split‐mouth clinical trial evaluated Sterify Gel as an adjunct to scaling and root planing and reported additional clinical improvements compared with mechanical therapy alone [13]. In addition, air‐polishing approaches using low‐abrasive powders such as glycine or erythritol have been investigated as adjunctive methods for biofilm management during periodontal therapy and supportive periodontal care [14, 15]. However, controlled trials and systematic reviews provide group‐level evidence under defined protocols and do not fully address the practical management of complex residual defects encountered in routine clinical practice.

The present case was considered clinically relevant because it involved a persistent deep residual pocket affecting a maxillary left first molar with buccal furcation involvement after initial nonsurgical periodontal therapy, in a highly compliant patient undergoing concurrent clear‐aligner orthodontic treatment. This clinical context creates both practical interest and important interpretive limitations. The observed outcome cannot be attributed to the hydrogel alone, because repeated mechanical instrumentation, erythritol‐based air polishing, plaque control, tissue maturation, and possible orthodontic influences may all have contributed to the clinical changes.

This case report is aimed at describing the clinical chronology, conservative retreatment protocol, short‐term outcomes, and limitations of a combined nonsurgical approach including repeated subgingival debridement, erythritol‐based air polishing, and adjunctive mucoadhesive polymeric hydrogel application for the management of a deep residual periodontal pocket with furcation involvement.

## 2. Materials and Methods

### 2.1. Patient Information and Baseline Periodontal Assessment

A 57‐year‐old systemically healthy, nonsmoking female patient was referred for comprehensive periodontal evaluation and treatment. Her medical history was unremarkable, with no reported systemic diseases, regular medications, allergies, diabetes mellitus, cardiovascular disease, osteoporosis, or other conditions known to influence periodontal disease progression or wound healing. The patient reported no history of tobacco use and no previous periodontal treatment other than routine professional prophylaxis. At the time of presentation, she was undergoing clear‐aligner orthodontic treatment, with aligners worn approximately 22 h per day.

A full‐mouth periodontal examination was performed using a UNC‐15 probe (Hu‐Friedy Manufacturing Co., Chicago, Illinois, United States). Probing depths were recorded at six sites per tooth to the nearest millimeter, and bleeding on probing was recorded dichotomously as present or absent within 15 s after probing. Plaque accumulation was assessed using the O′Leary Plaque Index. Baseline examination revealed generalized periodontal involvement, with probing depths ranging from 4 to 9 mm, bleeding on probing at 68% of sites, and a full‐mouth plaque score of 42%.

Radiographic assessment included panoramic radiography and periapical radiographs of the posterior maxillary region obtained using a digital sensor and film‐holder positioning system (Carestream CS 7200, Rochester, New York, United States, and Rinn XCP positioning system, Dentsply Sirona). Generalized horizontal alveolar bone loss was observed, with localized vertical bone loss in the left posterior maxilla, particularly in the interproximal region involving Tooth 26. Based on the 2017 World Workshop classification, the patient was diagnosed with periodontitis, Stage III Grade B [16]. Baseline radiographic and clinical documentation is shown in Figures 1, 2, 3, 4, and 5.

**Figure 1:**
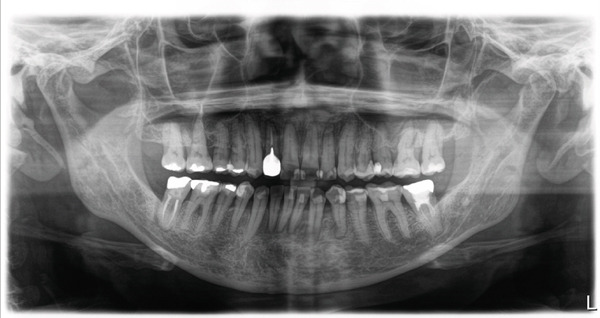
Baseline panoramic radiograph demonstrating generalized horizontal alveolar bone loss consistent with Stage III Grade B periodontitis. Severe vertical osseous defects are evident in the maxillary left posterior region, particularly in the interproximal areas between Teeth 25–26 and 26–27, where radiographic bone loss extends to approximately 50% of root length.

**Figure 2:**
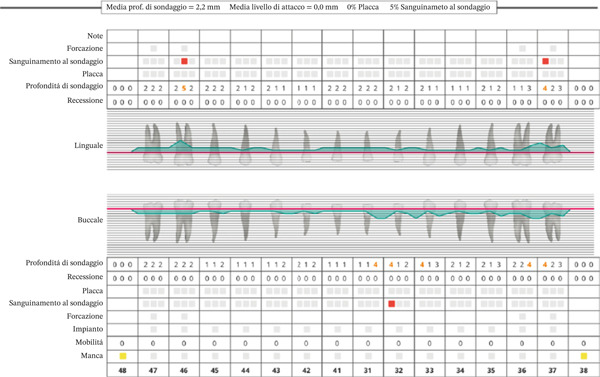
Periodontal charting of the mandibular arch at 3‐month re‐evaluation following initial comprehensive nonsurgical therapy. While overall probing depth reduction was achieved throughout most sites, isolated locations demonstrated incomplete response with residual probing depths of 4–5 mm at select positions.

**Figure 3:**
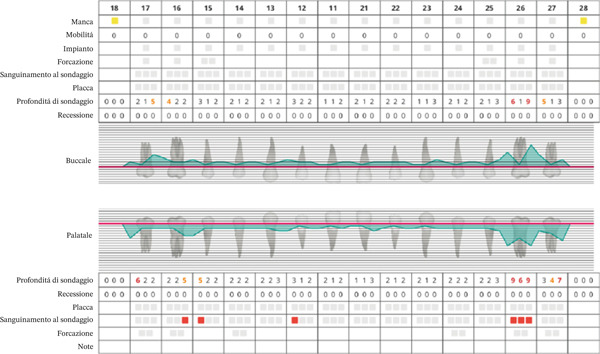
Periodontal charting of the maxillary arch at 3‐month re‐evaluation prior to adjunctive hydrogel therapy. The examination identified Tooth 26 exhibiting probing depths measuring 6 mm mesiopalatally and 9 mm distobuccally with profuse bleeding on probing, indicating localized failure to achieve therapeutic response despite excellent overall treatment outcomes and optimal patient plaque control (12% full‐mouth plaque score).

**Figure 4:**
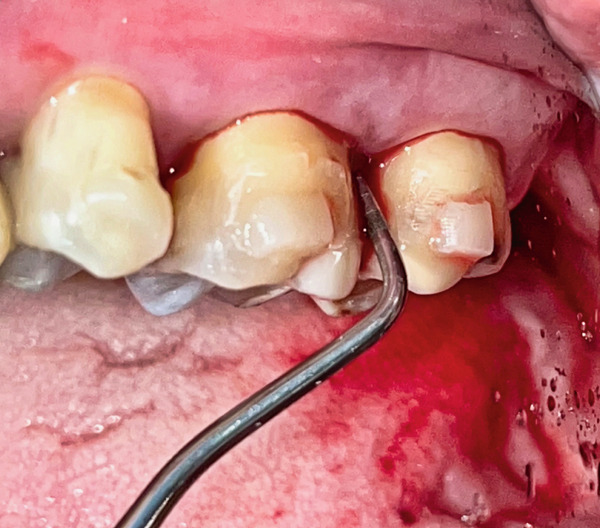
Clinical photograph demonstrating periodontal probing of the buccal aspect of Tooth 26 at baseline assessment prior to hydrogel application. The UNC‐15 periodontal probe penetrates to 9 mm at the distobuccal line angle, indicating a deep periodontal pocket with substantial clinical attachment loss and Grade II furcation involvement.

**Figure 5:**
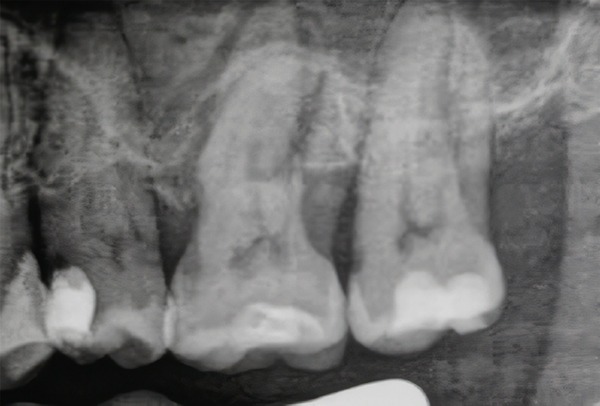
Baseline periapical radiograph of Teeth 25–27 demonstrating severe vertical alveolar bone loss. Radiolucency extends to approximately 50% of root length in the interproximal area between Teeth 25 and 26, representing a critical vertical osseous defect.

### 2.2. Initial Nonsurgical Periodontal Therapy and 3‐Month Re‐Evaluation

The patient underwent comprehensive nonsurgical periodontal therapy consisting of full‐mouth scaling and root planing completed over four appointments under local anesthesia (articaine hydrochloride 4% with epinephrine 1:100,000; Septodont, Saint‐Maur‐des‐Fossés, France). Treatment included hand instrumentation using Gracey curettes (Hu‐Friedy Manufacturing Co.) in combination with ultrasonic instrumentation (Piezon Master 700, EMS Electro Medical Systems, Nyon, Switzerland) with ultrasonic tips PS and PI, combined with individualized oral hygiene instructions. Instrumentation was performed under adequate illumination using LED headlights (3.5× magnification) to ensure visibility of subgingival anatomy. Oral hygiene instruction included toothbrushing and interdental cleaning techniques. A 0.12% chlorhexidine digluconate mouth rinse was prescribed for 14 days after completion of the initial nonsurgical periodontal therapy only. No chlorhexidine or other antiseptic rinse was prescribed after the subsequent hydrogel applications. The patient was enrolled in a periodontal maintenance program.

At the 3‐month re‐evaluation, the patient showed substantial improvement in plaque control and periodontal inflammation. The full‐mouth plaque score decreased from 42% to 12%, and bleeding on probing decreased from 68% to 28% of sites. Most periodontal sites showed clinical improvement; however, a localized residual defect persisted at Tooth 26, the maxillary left first molar. The tooth presented a 6 mm mesiopalatal probing depth and a 9 mm distobuccal probing depth, both with bleeding on probing. Buccal furcation involvement was classified as Grade II using a Nabers probe, according to the Hamp classification [17]. The periodontal charting and site‐specific clinical documentation at this time point are shown in Figures 2, 3, 4, and 5.

Treatment options, including periodontal surgery and conservative retreatment, were discussed with the patient. Considering the localized nature of the residual defect, the patient’s improved plaque control, absence of systemic risk factors, and preference to avoid surgery if possible, a conservative retreatment approach was selected. The selected approach consisted of repeated subgingival instrumentation, erythritol‐based air polishing, and adjunctive application of a mucoadhesive polymeric hydrogel.

### 2.3. Conservative Retreatment Protocol

The first conservative retreatment session was performed under local anesthesia. The involved sites at Tooth 26 were instrumented using periodontal curettes to access the root surfaces and furcation area. Subgingival erythritol‐based air polishing was then performed using a dedicated periodontal nozzle, with low‐abrasive erythritol powder delivered subgingivally according to the manufacturer′s instructions. This step was included as an adjunctive method for subgingival biofilm management, consistent with available evidence on low‐abrasive air‐polishing approaches in periodontal therapy and supportive periodontal care [14]. The pocket was subsequently irrigated with sterile saline solution.

Immediately after mechanical debridement and air polishing, a mucoadhesive polymeric hydrogel (Sterify Gel, Sterify Srl, Italy) was applied subgingivally using the prefilled syringe and blunt‐tip cannula supplied by the manufacturer. According to the available product documentation, the hydrogel consists of a polyvinyl alcohol/polyvinylpyrrolidone matrix containing nisin, hydroxytyrosol, and magnesium ascorbyl phosphate [15]. The cannula was inserted into the base of the pocket, and the gel was expressed slowly while withdrawing the cannula to fill the pocket from the apical to the coronal portion. Approximately 0.3 mL of hydrogel was applied. Gentle finger pressure was applied to the marginal tissues for approximately 30 s to promote adaptation of the material and reduce immediate extrusion. No customized stent was fabricated because the procedure was performed as part of routine clinical care rather than as a prospective research protocol; this limitation is addressed in the Discussion section.

Posttreatment instructions included avoiding eating or drinking for 1 h, gentle oral hygiene in the treated area for 24 h, resumption of regular oral hygiene after 24 h, and avoidance of hard or sticky foods on the treated side for 48 h. No antiseptic mouth rinse was prescribed after hydrogel application.

A clinical reassessment was performed 37 days after the first hydrogel application. The mesiopalatal probing depth decreased from 6 to 4 mm, and the distobuccal probing depth decreased from 9 to 5 mm; however, bleeding on probing persisted. Therefore, a second application of the hydrogel was performed after gentle subgingival biofilm removal with erythritol‐based air polishing and saline irrigation. The same application technique and approximate volume were used. The first retreatment session, air‐polishing step, hydrogel application, interim assessment, and second application are documented in Figures 6, 7, 8, 9, 10, and 11.

**Figure 6:**
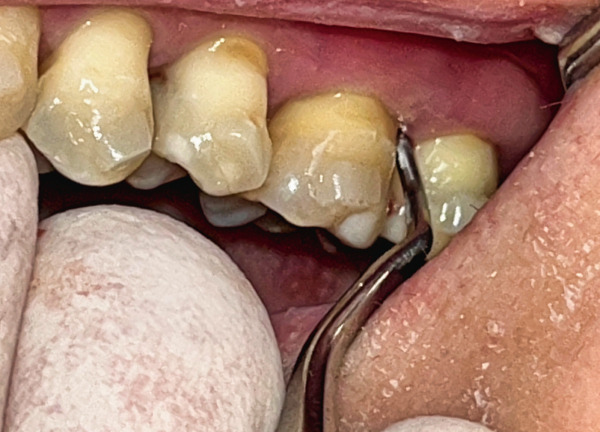
Subgingival instrumentation using periodontal curettes during the conservative retreatment session. The curette design provides appropriate blade angulation for accessing the complex root anatomy of the maxillary first molar, including furcation areas.

**Figure 7:**
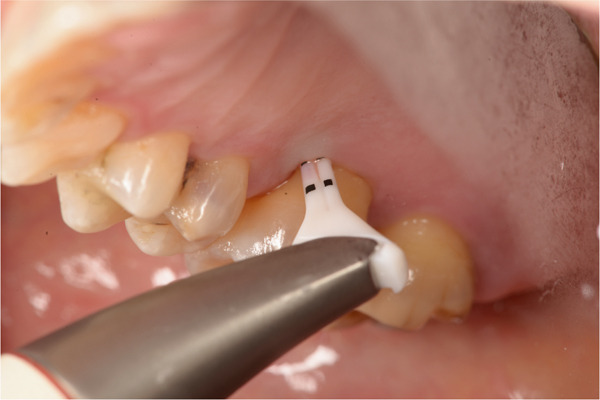
Application of erythritol‐based air polishing using a specialized subgingival delivery nozzle (Air‐Flow Perio system). The system delivers erythritol powder (14 *μ*m particle size) suspended in warm water at controlled low pressure (5 bar) to accomplish gentle biofilm removal without aggressive mechanical tissue disruption.

**Figure 8:**
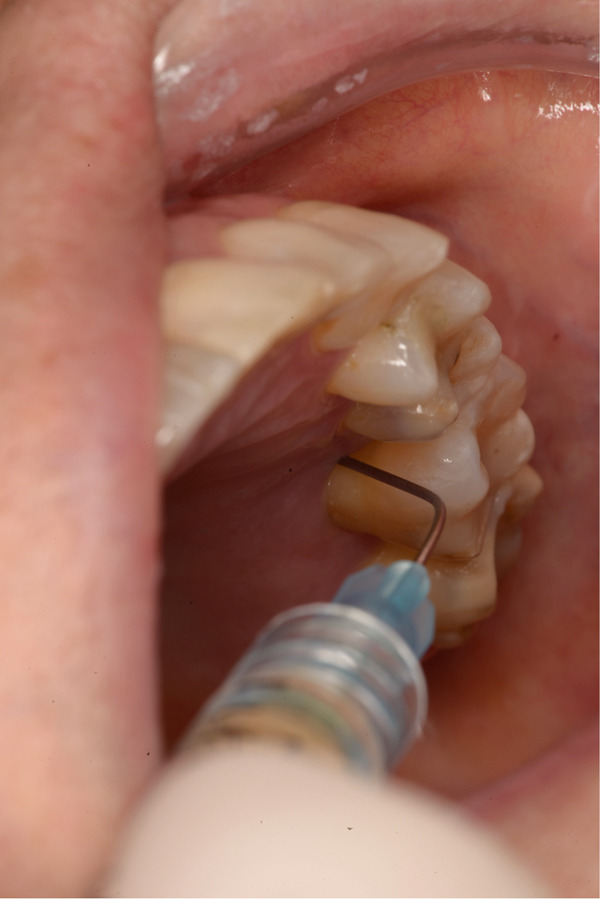
Subgingival delivery of the mucoadhesive polymeric hydrogel (Sterify Gel) into the periodontal pocket immediately following comprehensive mechanical debridement. The prefilled syringe delivery system with blunt‐tip cannula permits controlled placement of the polyvinyl polymer‐based gel to full pocket depth.

**Figure 9:**
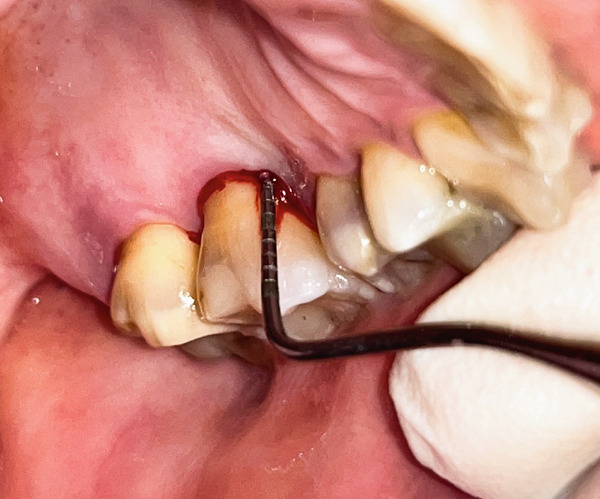
Clinical photograph at 37‐day interim assessment demonstrating periodontal probing of the mesiopalatal aspect of Tooth 26. The probing depth decreased from baseline 6 to 4 mm at this evaluation time point, demonstrating early clinical improvement though bleeding on probing remained present.

**Figure 10:**
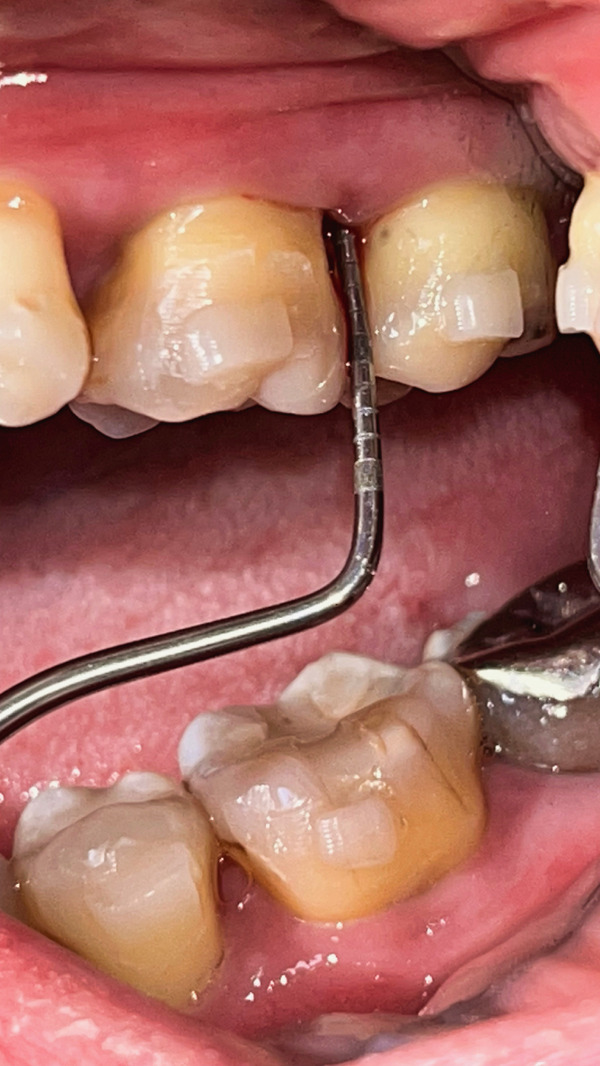
Clinical photograph at 37‐day interim assessment showing probing of the distobuccal aspect of Tooth 26. The probing depth decreased from the initial 9 to 5 mm, representing substantial though incomplete pocket reduction, with persistent bleeding on probing indicating continued inflammation.

**Figure 11:**
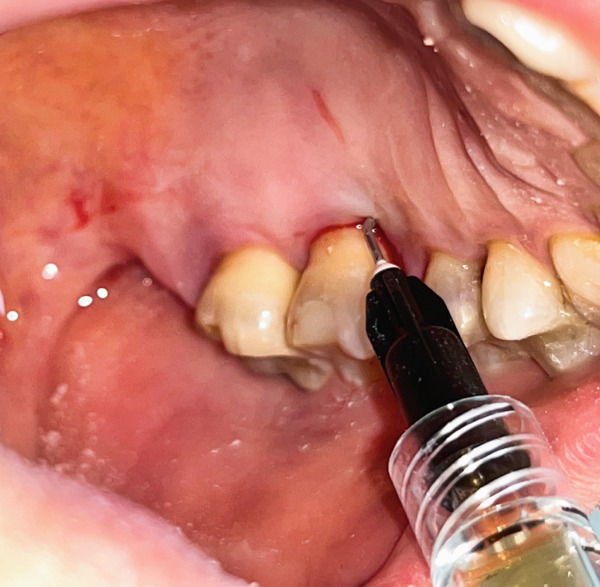
Second application of mucoadhesive polymeric hydrogel at the 37‐day interim assessment following subgingival biofilm removal with erythritol‐based air polishing. The decision to perform a second application was based on partial clinical response (pocket depth reduction but persistent inflammation) observed at this time point.

### 2.4. Outcome Assessment and Follow‐Up

Clinical outcomes were assessed at baseline, at the 3‐month re‐evaluation after initial nonsurgical periodontal therapy, 37 days after the first hydrogel application, and 4 months after the first hydrogel application. At each relevant time point, probing depth, bleeding on probing, plaque control, and furcation involvement were recorded. The primary site‐specific outcomes were probing depth reduction, bleeding on probing resolution, and change in furcation classification at Tooth 26.

Periapical radiographs were obtained at baseline and at 4‐month follow‐up using a standardized film‐holder technique and comparable exposure settings. Radiographic changes were assessed by visual comparison only. Digital subtraction radiography, cone‐beam computed tomography, and histologic confirmation were not performed; therefore, radiographic findings were interpreted cautiously and were not considered definitive evidence of new bone formation.

Clinical photographs were obtained during routine clinical documentation. Because the photographs were not acquired under a prospective research photography protocol, variability in image quality, angulation, and standardization was possible. Clinical measurements, rather than photographic appearance alone, were therefore considered the main basis for outcome assessment.

### 2.5. Ethical Considerations

This case report describes retrospective documentation of routine clinical care using commercially available materials according to clinical indications and manufacturer instructions. The patient provided written informed consent for publication of anonymized clinical data, clinical photographs, and radiographic images for educational and scientific purposes. All identifiable information was removed to protect patient privacy. Because this was a single anonymized case report based on routine care and written informed consent was obtained, formal ethics committee approval was not required according to local requirements. The report was prepared in accordance with the principles of the Declaration of Helsinki and the CARE guidelines.

## 3. Results

### 3.1. Response to Initial Nonsurgical Periodontal Therapy

At the 3‐month re‐evaluation after initial nonsurgical periodontal therapy, the patient showed marked improvement in full‐mouth plaque control and periodontal inflammation. The full‐mouth plaque score decreased from 42% at baseline to 12%, and bleeding on probing decreased from 68% to 28% of sites.

Most periodontal sites showed clinical improvement after the initial therapy. However, a localized residual defect persisted at Tooth 26, the maxillary left first molar. At this time point, the mesiopalatal probing depth was 6 mm, and the distobuccal probing depth was 9 mm, both associated with bleeding on probing. Buccal furcation involvement was classified as Grade II. These findings identified Tooth 26 as the main residual site requiring further treatment. The 3‐month periodontal charting and site‐specific clinical documentation are shown in Figures 2, 3, 4, and 5.

### 3.2. Response After the First Hydrogel Application

At 37 days after the first combined conservative retreatment session, partial clinical improvement was observed at Tooth 26. The mesiopalatal probing depth decreased from 6 to 4 mm, and the distobuccal probing depth decreased from 9 to 5 mm. However, bleeding on probing was still present at the treated sites.

No adverse effects, posttreatment complications, or patient‐reported discomfort were recorded during this interval. The patient maintained good plaque control and attended the scheduled follow‐up visit. Because pocket reduction was observed but inflammation had not fully resolved, a second hydrogel application was performed after gentle subgingival biofilm removal. The interim clinical findings and second application are shown in Figures 9, 10, and 11.

### 3.3. Four‐Month Clinical Outcomes

At 4 months after the first hydrogel application, further clinical improvement was observed at Tooth 26. The mesiopalatal probing depth measured 2 mm, corresponding to a 4 mm reduction from the preretreatment value of 6 mm. The distobuccal probing depth measured 3 mm, corresponding to a 6 mm reduction from the preretreatment value of 9 mm. Bleeding on probing was absent at the previously involved sites.

The buccal furcation involvement was clinically reduced from Grade II to Grade I. The gingival tissues appeared clinically stable, with no suppuration, swelling, or adverse soft‐tissue reaction. Full‐mouth plaque control remained stable, with plaque scores maintained below 15%, and no new deep periodontal pockets were detected during the observation period. The 4‐month periodontal charting and clinical documentation are shown in Figures 12, 13, 14, 15, and 16.

**Figure 12:**
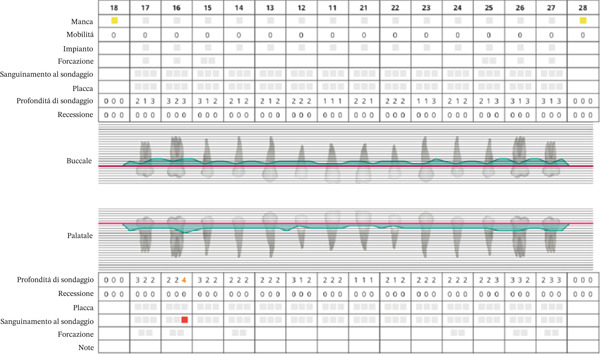
Periodontal charting of the mandibular arch at 4‐month follow‐up evaluation demonstrating restoration of physiological probing depths (≤ 3 mm) at nearly all measured sites with minimal bleeding on probing, indicating maintenance of periodontal health achieved through initial comprehensive nonsurgical therapy.

**Figure 13:**
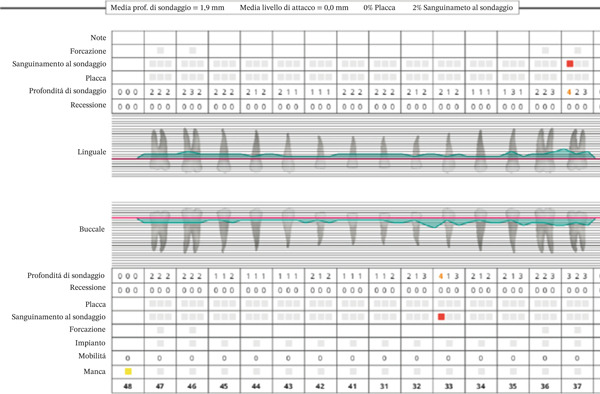
Periodontal charting of the maxillary arch at 4‐month follow‐up evaluation revealing resolution of previously deep pockets throughout the dentition. Probing depths of ≤ 3 mm predominated with complete absence of bleeding on probing at the previously compromised sites, including Tooth 26 which exhibited 2 mm mesiopalatally and 3 mm distobuccally.

**Figure 14:**
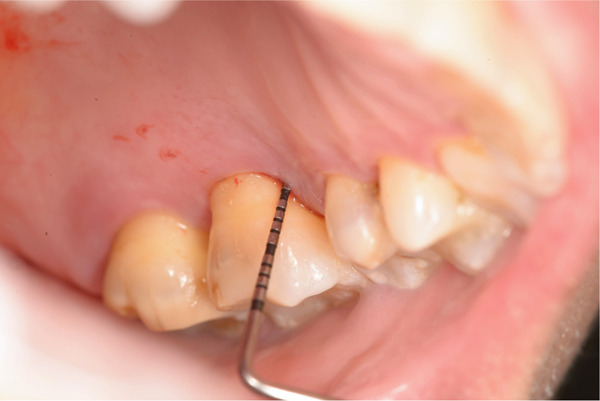
Clinical photograph of the buccal aspect of Tooth 26 at 4‐month follow‐up demonstrating periodontal probing. The previously compromised distobuccal site now exhibited a physiological probing depth of 3 mm with complete absence of bleeding on probing and healthy gingival tissue appearance.

**Figure 15:**
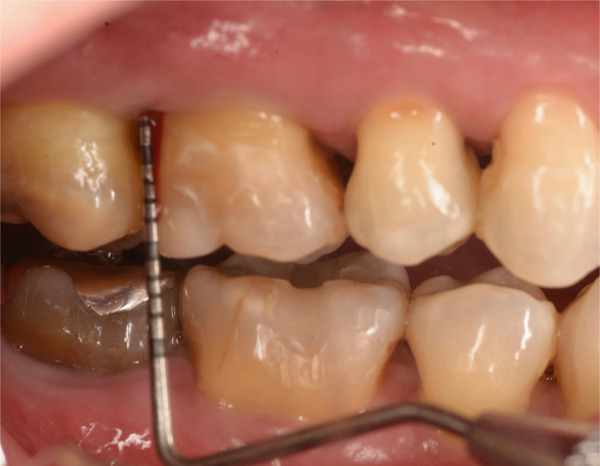
Clinical photograph demonstrating probing of Tooth 26 from the palatal aspect at 4‐month follow‐up evaluation. Probing depths measured 2 mm mesiopalatally with healthy coral‐pink gingival tissue and complete absence of bleeding, indicating successful treatment outcome.

**Figure 16:**
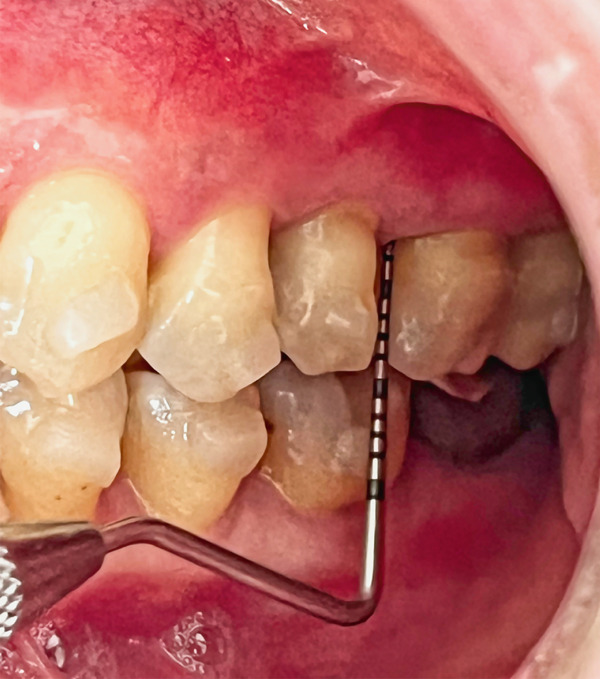
Clinical photograph demonstrating overall gingival tissue appearance in the maxillary left posterior region at 4‐month follow‐up evaluation. The gingival tissues exhibit healthy coral‐pink coloration, firm consistency, and good adaptation to tooth surfaces, consistent with successful conservative periodontal therapy outcome.

The patient reported satisfaction with the conservative treatment approach and did not report pain, discomfort, or functional limitations related to the treated site during follow‐up. No adverse events were recorded.

### 3.4. Radiographic Observations

Visual comparison of baseline and 4‐month periapical radiographs showed increased radiopacity in the interproximal area between Teeth 25 and 26, particularly in the coronal portion of the previously radiolucent defect (Figure 17). This finding was interpreted cautiously as a radiographic observation compatible with possible mineralized tissue changes or remodeling.

**Figure 17:**
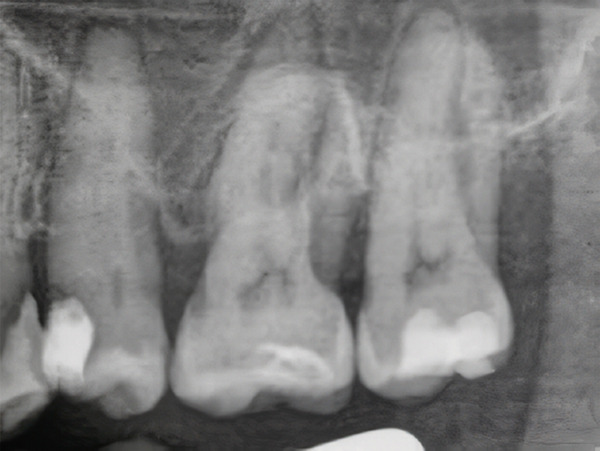
Four‐month follow‐up periapical radiograph of Teeth 25–27 demonstrating increased bone radiopacity in the interproximal area between Teeth 25 and 26 compared to baseline radiographic appearance. This finding suggests possible new bone formation, increased mineralization of existing trabecular bone, or combination thereof, though definitive confirmation would require histologic examination. Interpretation is limited by potential geometric variations in radiographic angulation and absence of digital subtraction radiography.

Because the radiographs were assessed by visual comparison only and because digital subtraction radiography, cone‐beam computed tomography, and histologic confirmation were not performed, this radiographic change was not considered definitive evidence of new bone formation. The radiographic observation was therefore interpreted as supportive but secondary to the clinical outcomes.

## 4. Discussion

### 4.1. Treatment Outcomes in Clinical Context

This case report describes the short‐term clinical improvement of a deep residual periodontal pocket with buccal furcation involvement at Tooth 26, the maxillary left first molar, after a combined conservative retreatment protocol. The site had shown an incomplete response after initial nonsurgical periodontal therapy despite improved plaque control and general periodontal improvement. After repeated subgingival instrumentation, erythritol‐based air polishing, and two adjunctive applications of a mucoadhesive polymeric hydrogel, the distobuccal probing depth decreased from 9 to 3 mm, bleeding on probing resolved, and buccal furcation involvement was clinically reduced from Grade II to Grade I.

These findings are clinically relevant because residual deep pockets after subgingival instrumentation may remain difficult to maintain and may represent sites at higher risk for disease progression, especially in molars with complex root anatomy and furcation involvement [3–6, 17]. However, the outcome must be interpreted cautiously. This is a single case report with a short follow‐up period and without a control site. Therefore, the present report cannot establish treatment efficacy, causality, or equivalence to periodontal surgery. The case should instead be interpreted as a documented example of short‐term improvement associated with a combined conservative retreatment approach in a highly compliant patient.

### 4.2. Relationship With Existing Evidence on Local Adjunctive Therapies

Local adjunctive therapies have been investigated to improve periodontal outcomes after mechanical instrumentation, particularly in sites where access, residual biofilm, and persistent inflammation may limit the response to subgingival debridement alone [7, 8, 18]. Traditional locally delivered antimicrobials and antiseptics have generally shown modest additional benefits when compared with mechanical therapy alone [8, 18]. More recent approaches, including hydrogels and other bioactive delivery systems, are aimed at combining local retention with the delivery of active components within the periodontal pocket [9–12, 19–21].

The randomized split‐mouth clinical trial evaluating Sterify Gel as an adjunct to scaling and root planing provides higher level evidence than the present report and supports the potential clinical relevance of this material as an adjunctive therapy [13]. In addition, product‐related microbiological documentation has suggested reductions in selected periodontal pathogens after treatment with this formulation [15]. Nevertheless, these data do not allow direct attribution of the present case outcome to the hydrogel alone. The clinical improvement observed here occurred within a broader protocol that included repeated instrumentation, air polishing, improved plaque control, and follow‐up monitoring.

The specific formulation used in this case includes a polyvinyl alcohol/polyvinylpyrrolidone matrix with nisin, hydroxytyrosol, and magnesium ascorbyl phosphate. From a mechanistic perspective, the polymeric matrix may contribute to local retention and mucoadhesion, while the active components have been associated in experimental or biomedical literature with antimicrobial, antioxidant, anti‐inflammatory, and extracellular matrix‐related effects [19–29]. These mechanisms remain biologically plausible but should not be overinterpreted in a single clinical case. No microbiological, biomarker, histologic, or controlled comparative assessment was performed in this patient.

### 4.3. Role of Erythritol‐Based Air Polishing Within the Combined Protocol

Subgingival erythritol‐based air polishing was included as part of the conservative retreatment protocol to support biofilm disruption after mechanical instrumentation. Low‐abrasive air‐polishing powders such as glycine and erythritol have been investigated in periodontal therapy and supportive periodontal care, with the aim of improving subgingival biofilm management while limiting hard‐ and soft‐tissue trauma [14]. In the present case, air polishing should be considered a component of the combined retreatment protocol rather than a secondary or negligible procedural detail.

This point is important for interpretation. The observed clinical improvement cannot be assigned specifically to the hydrogel because subgingival air polishing, repeated mechanical debridement, and improved plaque control may each have contributed to the reduction in inflammation and probing depth. The present report therefore supports the feasibility of a combined conservative approach, but it does not isolate the independent effect of any single component.

### 4.4. Furcation and Radiographic Interpretation

The clinical change in buccal furcation involvement from Grade II to Grade I was recorded as a site‐specific finding in this patient. This observation may reflect reduced inflammation, decreased probing penetration, soft‐tissue adaptation, and/or changes in local access for probing. It should not be interpreted as evidence that the hydrogel has a specific furcation‐related therapeutic effect. Available evidence on the hydrogel does not establish a furcation‐specific benefit, and no controlled furcation‐focused assessment was performed in the present case [13, 17].

Similarly, the increased radiopacity observed between Teeth 25 and 26 at 4 months should be interpreted with caution. Periapical radiographs obtained in routine clinical care may be affected by differences in angulation, projection geometry, sensor positioning, and image density. Without digital subtraction radiography, cone‐beam computed tomography, or histologic confirmation, radiographic changes cannot be considered definitive evidence of new bone formation. The radiographic finding is therefore secondary to the clinical outcomes and should be described only as a visual observation compatible with possible mineralized tissue change or remodeling.

### 4.5. Comparison With Surgical and Adjunctive Approaches

Surgical access procedures and regenerative approaches remain established options for residual deep pockets and intrabony defects when indicated [30, 31]. Open flap debridement may improve access for instrumentation, while regenerative procedures may be considered in selected defects with favorable anatomy [30, 31]. The present case does not demonstrate that conservative retreatment can replace surgical therapy. Rather, it documents that short‐term clinical improvement was observed after a nonsurgical combined protocol in a patient with good plaque control and a preference to avoid surgery.

The clinical context is therefore important. Conservative retreatment may be reasonable to consider before surgery in selected patients when inflammation control, plaque control, and careful monitoring are achievable. However, persistent bleeding, recurrent deep probing depths, suppuration, progressive attachment loss, or radiographic progression would still require reassessment and possible escalation of therapy. Local antimicrobials, host‐modulation approaches, photodynamic therapy, and probiotics have also been investigated as adjunctive strategies, but their effects vary and should be considered adjunctive to, not replacements for, mechanical biofilm control and supportive periodontal care [32–36].

### 4.6. Limitations

The main limitation of this report is its single‐case design. Without randomization, a control site, or blinded outcome assessment, causal inference is not possible. The specific contribution of the hydrogel cannot be separated from repeated mechanical instrumentation, erythritol‐based air polishing, improved oral hygiene, natural tissue maturation after initial periodontal therapy, and professional follow‐up. The patient’s high compliance and absence of major systemic or smoking‐related risk factors also limit generalizability to broader periodontal populations.

Concurrent clear‐aligner orthodontic treatment represents an additional confounding factor. The aligners may theoretically have influenced local plaque control, tissue maturation, occlusal loading, tooth position, or hydrogel retention, although they were not designed or fabricated as periodontal stents. No customized stent was used either for hydrogel retention or for standardized probing. This reflects the routine‐care nature of the treatment but limits measurement standardization and should be considered when interpreting probing depth changes.

The follow‐up period was limited to 4 months after the first hydrogel application. This interval is sufficient to describe early clinical changes but not long‐term stability. Periodontal recurrence or further improvement cannot be assessed without longer follow‐up. In addition, no microbiological sampling, inflammatory biomarker assessment, digital subtraction radiography, cone‐beam computed tomography, or histologic analysis was performed. The report was also based on routine clinical photographs rather than a prospective standardized photographic protocol.

A further limitation is that several biological mechanisms proposed for hydrogel‐based systems derive from experimental, material‐science, or general biomedical studies rather than from direct clinical evidence in furcation‐involved residual periodontal pockets [18–29, 37]. These mechanisms may help explain biological plausibility, but they do not prove clinical efficacy in the present case. Future studies should evaluate these adjunctive approaches using controlled designs, standardized probing methods, longer follow‐up, and clearly defined clinical, radiographic, microbiological, and patient‐reported outcomes.

### 4.7. Clinical Relevance of the Case

Despite these limitations, the present case has practical value because it documents the management of a complex residual molar site that may be encountered in routine periodontal care: a persistent deep pocket with furcation involvement after otherwise effective nonsurgical therapy. The report also illustrates the importance of describing adjunctive materials within the full clinical context in which they are used. In this patient, the outcome was associated with a combined conservative protocol, high plaque‐control compliance, careful reassessment, and repeated professional intervention.

The main clinical message is therefore not that hydrogel therapy is an alternative to periodontal surgery, but that selected residual pockets may show short‐term improvement after a structured conservative retreatment protocol. Such an approach should be considered descriptive and hypothesis‐generating rather than confirmatory, as is appropriate for single‐case clinical reporting [38, 39]. Long‐term supportive periodontal care and continued monitoring remain necessary to determine whether the observed clinical improvements are stable over time.

## 5. Conclusions

This case report describes short‐term clinical improvement of a deep residual periodontal pocket with buccal furcation involvement at Tooth 26, the maxillary left first molar, after a combined conservative retreatment protocol including repeated subgingival instrumentation, erythritol‐based air polishing, and adjunctive mucoadhesive polymeric hydrogel application. At 4 months, probing depth was reduced, bleeding on probing was absent, and buccal furcation involvement was clinically reduced from Grade II to Grade I.

Because this was a single case without a control site, the specific contribution of the hydrogel cannot be isolated from repeated mechanical debridement, air polishing, plaque control, tissue maturation, and possible effects of concurrent clear‐aligner therapy. The radiographic findings should also be interpreted cautiously and cannot be considered definitive evidence of new bone formation.

The present case should therefore be considered descriptive and hypothesis‐generating. In selected highly compliant patients, a structured conservative retreatment approach may be considered before surgical escalation, provided that careful clinical monitoring and supportive periodontal care are maintained. Controlled studies with longer follow‐up are needed to clarify the efficacy, indications, and long‐term stability of this combined approach.

## Author Contributions

Conceptualization: Stefano Sarri and Simone Sevi. Methodology: Stefano Sarri and Riccardo Scaringi. Investigation: Stefano Sarri and Riccardo Scaringi. Resources: Stefano Sarri. Data curation: Stefano Sarri, Lorenzo De Marco, and Riccardo Scaringi. Formal analysis: Simone Sevi. Visualization: Simone Sevi and Lorenzo De Marco. Writing—original draft preparation: Simone Sevi. Writing—review and editing: Stefano Sarri, Scilla Sparabombe, Riccardo Scaringi, Enrico Fiorini, Lorenzo De Marco, and Simone Sevi. Supervision: Scilla Sparabombe and Enrico Fiorini. Project administration: Simone Sevi.

## Funding

Open access publishing facilitated by Universita degli Studi dell’Insubria, as part of the Wiley ‐ CRUI‐CARE agreement.

## Disclosure

All authors have read and approved the final version of the manuscript. Simone Sevi, as corresponding author and manuscript guarantor, had full access to all of the data in this study and takes complete responsibility for the integrity of the data and the accuracy of the data analysis. The product used in this case was commercially available and was used according to clinical indications. The authors verified the cited references for retractions and published corrections using PubMed and reference‐manager checks. No cited article was identified as retracted at the time of resubmission. Any bibliographic discrepancies identified during revision were corrected and did not affect the interpretation of the cited evidence.

## Ethics Statement

This case report describes a single anonymized patient treated as part of routine clinical care. Written informed consent was obtained for publication of anonymized clinical data, clinical photographs, and radiographic images. Because this was a retrospective single‐case report based on routine care, with full anonymization and written informed consent, formal ethics committee approval was not required according to local requirements. The report was prepared in accordance with the principles of the Declaration of Helsinki and the CARE guidelines.

## Consent

Written informed consent was obtained from the patient for publication of this case report and accompanying images in anonymized form. The patient consented to the use of deidentified clinical data, photographs, and radiographic images for educational and research publication purposes. A copy of the written consent is available for review by the editor of this journal.

## Conflicts of Interest

The authors declare no conflicts of interest.

## Data Availability

The authors confirm that the data supporting the findings of this study are available within the article and/or its Supporting Information. Additional anonymized information may be made available by the corresponding author upon reasonable request, where compatible with patient privacy and informed consent.
